# Parent-child nature visits and young Finnish childreńs nature connectedness: Carola Ray

**DOI:** 10.1093/eurpub/ckac131.336

**Published:** 2022-10-25

**Authors:** C Ray, J Kokkonen, N Simonsen, N Wackström, J Ray, E Engberg

**Affiliations:** 1 Public Health, Folkhälsan Research Center, Helsinki, Finland; 2 Department of Food and Nutrition, University of Helsinki, Helsinki, Finland; 3 Department of Public Health, University of Helsinki, Helsinki, Finland; 4 Department of Psychology and Logopedics, University of Helsinki, Helsinki, Finland

## Abstract

**Background:**

It is well-known that frequent nature visits are associated with health benefits in children. Global climate crisis and environmental challenges emphasize the need for reconnecting children to nature, as well. Young children’s nature connectedness (NC) involves enjoyment of nature, sense of responsibility, empathy, and awareness of nature. The study examines whether there is an association between the frequency of parent-child nature visits and young children’s NC.

**Methods:**

The study uses WEB survey data from the Finnish Empowered by Nature project. Respondents, n = 1463, were parents of children aged 2 to 7 years old. NC was assessed by 11-items derived from the NC questionnaire of Sobko et al (2018). Multinomial logistic regression analysis was used and the lowest tertile of NC was used as reference group. Analysis were adjusted for child’s age and gender, and highest education of parent.

**Results:**

Children with moderate (1-2 times a week in previous month) or high (3 times a week or more) frequency of parent-child nature visits were more likely to have strong than weak NC compared to children with low frequency (less than once a week) of adult-child nature visits. Odds ratios (OR) in adjusted models were: moderate frequency 1.67 (1.21- 2.32), and high frequency 2.31 (1.67-3.18). The odds of having medium NC compared to weak NC were more likely in moderate frequency compared to low frequency of parent-child nature visits in the adjusted model (OR 1.46; 1.07-2.00).

**Conclusions:**

The results highlight the importance of parents visiting nature frequently with their children during early childhood. It promotes young children’s NC and may further contribute to raising environmentally responsible children.

**Key messages:**

• More frequent parent-child visits promote young children’s nature connectedness which involves enjoyment of nature, sense of responsibility, empathy, and awareness of nature.

• A strong nature connectedness among children is highly relevant, as it may contribute to raise environmentally responsible children.

